# Bloch Surface Wave Resonance Based Sensors as an Alternative to Surface Plasmon Resonance Sensors

**DOI:** 10.3390/s20185119

**Published:** 2020-09-08

**Authors:** Michal Gryga, Dalibor Ciprian, Petr Hlubina

**Affiliations:** Department of Physics, Technical University Ostrava, 17. listopadu 2172/15, 708 00 Ostrava-Poruba, Czech Republic; michal.gryga@vsb.cz (M.G.); dalibor.ciprian@vsb.cz (D.C.)

**Keywords:** Bloch surface wave resonance, surface plasmon resonance, Kretschmann configuration, multilayer plasmonic structure, multilayer dielectric structure, spectral interference, moist air, sensitivity, figure of merit

## Abstract

We report on a highly sensitive measurement of the relative humidity (RH) of moist air using both the surface plasmon resonance (SPR) and Bloch surface wave resonance (BSWR). Both resonances are resolved in the Kretschmann configuration when the wavelength interrogation method is utilized. The SPR is revealed for a multilayer plasmonic structure of SF10/Cr/Au, while the BSWR is resolved for a multilayer dielectric structure (MDS) comprising four bilayers of TiO${}_{2}$/SiO${}_{2}$ with a rough termination layer of TiO${}_{2}$. The SPR effect is manifested by a dip in the reflectance of a *p*-polarized wave, and a shift of the dip with the change in the RH, or equivalently with the change in the refractive index of moist air is revealed, giving a sensitivity in a range of 0.042–0.072 nm/%RH. The BSWR effect is manifested by a dip in the reflectance of the spectral interference of *s*- and *p*-polarized waves, which represents an effective approach in resolving the resonance with maximum depth. For the MDS under study, the BSWRs were resolved within two band gaps, and for moist air we obtained sensitivities of 0.021–0.038 nm/%RH and 0.046–0.065 nm/%RH, respectively. We also revealed that the SPR based RH measurement is with the figure of merit (FOM) up to 4.7 × 10^−4^ %RH${}^{-1}$, while BSWR based measurements have FOMs as high as 3.0 × 10${}^{-3}$ %RH${}^{-1}$ and 1.1 × 10^−3^ %RH${}^{-1}$, respectively. The obtained spectral interferometry based results demonstrate that the BSWR based sensor employing the available MDS has a similar sensitivity as the SPR based sensor, but outperforms it in the FOM. BSW based sensors employing dielectrics thus represent an effective alternative with a number of advantages, including better mechanical and chemical stability than metal films used in SPR sensing.

## 1. Introduction

Surface plasmon resonance (SPR) based optical sensors as the heart of a mature technology in the field of optical sensing have a number of applications in physics [1,2], chemistry [3], biology [4], and other fields [5,6,7]. The SPR sensors utilize the interaction of light with free electrons at a metal-dielectric interface [8], and thus are very sensitive to the refractive index changes in a very thin layer at the sensor surface. To attain the resonance, surface plasmons (SPs) representing the collective oscillations of free electrons are excited at the interface by a technique of the attenuated total reflection (ATR). As an example, the SPs are generated in the Kretschmann configuration [1] employing a high refractive index prism coated on its base by a thin metal film, and the SPs are excited in the metal film by the ATR mechanism. The optical field of the SPs decays exponentially on both sides of the dielectric/metal interface. Consequently, the SPR phenomenon [4,9] related to the generation of the surface plasmon polaritons (SPPs) and propagation of the surface plasmon wave (SPW) along the dielectric/metal interface, is accompanied by a drop in the reflected intensity [4], or by a phase change [10,11], and thus it is attractive to sensing in various fields of interest [12,13,14,15,16,17,18,19,20,21,22,23,24,25], especially in biosensing [4,15,16,17,18]. To these measurements, performed in angular or spectral domain, correspond angular or wavelength interrogations. When the angular interrogation [4,5,6] with a monochromatic beam is considered, a sharp minimum (dip) is resolved in the angular spectrum. Similarly, when the wavelength interrogation [19,20,21,22,26] with a white-light source is considered, a dip is resolved in the reflection spectrum. In addition, a large number of fiber optic sensors utilizing the SPR have been proposed and realized as those presented in review papers [27,28,29,30] or in [31,32].

Surface waves which are confined to the interface between a homogeneous medium and a multilayer dielectric structure (MDS) [33] such as a finite one-dimensional photonic crystal (1DPhC) or Bragg mirror have found a large number of applications similar to the SPPs [34,35,36,37,38,39,40,41,42,43,44,45,46,47,48,49,50,51,52,53]. Light confinement in the surface waves, which are referred to as Bloch surface waves (BSWs), occurs near the multilayer surface and it is caused by the total internal reflection (TIR) from the homogeneous layer. Because dispersion of the BSWs is located within the forbidden bands (band gaps) of the 1DPhC, exponential decays of both the field envelope inside the 1DPhC and the field in the homogeneous medium are present. When a metal and a dielectric Bragg mirror are considered, surface waves referred to as Tamm plasmons (TPs) can be revealed [54]. Contrary to the SPPs, the TPs can be optically excited by both *s*- and *p*-polarized waves, and a direct excitation from free space is possible [55,56,57] as well.

Comparing BSWs to SPPs, several important differences and advantages can be identified. Contrary to SPPs, the BSWs can be excited by both *s*- and *p*-polarized waves [44] at any wavelength, simply by changing the geometry and materials of the photonic crystal. The BSW based sensors enable sharper resonances than conventional SPR sensors because they do not rely on the use of metals. Because of the dielectric materials of the photonic crystal, sensors based on BSWs are characterized by mechanical and chemical stability, thus enabling operation in aggressive environments. Consequently, BSWs have emerged as an effective alternative to SPPs [43,58] and have been utilized in a number of BSW based sensors [37,38,39,40,41,42,45,46,47,48,49,50,51,52,53,59,60,61,62,63,64,65,66], especially in biosensing [37,38,39,40,41,42]. Similarly to SPR based sensors, Bloch surface wave resonance (BSWR) based sensing is with a special emphasis on the measured quantity such as phase [46,47,48,49] or intensity [50,51,52,53,59,60,61,62,63]. In addition, the angular [47,48,49,50,51,52,53,60] or the wavelength interrogations [46,61,62,63,64,65,66] have been employed. Similarly to the SPR based fiber optic sensors, fiber sensors with MDSs have been reported [67,68,69,70], and MDSs have been deposited on a tapered fiber [67] or on the outer surface of optical fiber [68]. Moreover, configurations with the MDSs deposited at the tip of a single-mode fiber [69] or inside of a photonic crystal fiber [70] have been considered.

In this paper, we present results of a highly sensitive measurement of the relative humidity (RH) of moist air using the SPR and BSWR, and employing the Kretschmann configuration, when a shift in the resonance is affected by a change in the refractive indices of the external medium (moist air) of the sensor and its termination material. The resonances are resolved for a multilayer plasmonic structure (MPS) of SF10/Cr/Au, and for an MDS comprising four bilayers of TiO${}_{2}$/SiO${}_{2}$ with a rough termination layer of TiO${}_{2}$ using a spectral interrogation method. In the measurements, the shift of both the SPR and BSWR with the varied RH, or equivalently with the varied refractive index of air, is determined from the shift of the dip of the reflectance ratio. For the SPR, we measure the ratio of reflectances of *p*- and *s*-polarized waves. For the BSWR, we use a different approach [63] in resolving the resonance with maximum depth and measure the ratio of reflectances of two waves, when one wave is the result of the spectral interference of *s*- and *p*-polarized waves and the other one is the polarized wave for which the BSWR does not occur. The obtained spectral interferometry based results demonstrate that the BSWR based sensor has a similar sensitivity as the SPR based sensor, but outperforms it in the figure of merit (FOM). Compared to the SPR based sensors employing metal films such as gold or silver, which are quickly degraded in aggressive environments, BSW based sensors employing dielectrics thus represent an effective alternative with advantages such as better mechanical and chemical stability.

## 2. Background

### 2.1. Optical Response Computation Method

The current trend in the sensing approaches prefers to utilize the wavelength interrogation and so the reflectance responses computation of the SPR and BSWR based systems is desirable. As in both of the mentioned cases, one deals with thin film structures, the transfer matrix method (TMM), often used in optics of layered media [71,72], is used in most cases [73]. The goal is to obtain the reflection and transmission coefficients, or solve the dispersion equation of the structure. To express the reflectances for *p*- and *s*-polarized waves, $R_{p}\left(\lambda \right)$ and $R_{s}\left(\lambda \right)$, as a function of the wavelength λ, we evaluate a total transmission matrix of a multilayer structure of known optical characteristics using a standard approach [61].

### 2.2. SPR Structure

In order to explain the results obtained using the TMM, the dispersion equation, which relates the wave vector of surface waves to its frequency (or wavelength), is needed. Considering the case of the SPW, we have the equation

**Figure 1:**
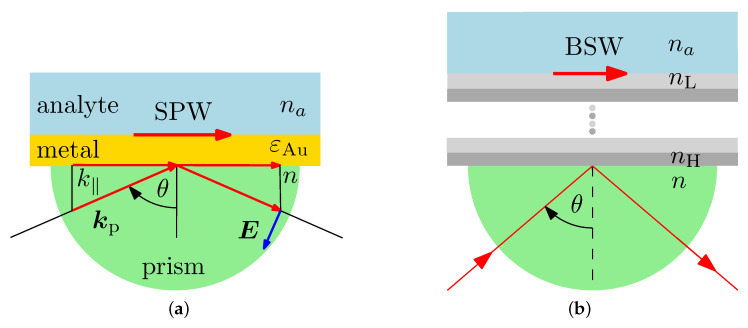
The Kretschmann configuration: coupling of an optical wave into the SPW (**a**) and the BSW, respectively (**b**).

(1) kspw(λ)=2πλReε1(λ)ε2(λ)ε1(λ)+ε2(λ).

where $k_{\mathrm{spw}}\left(\lambda \right)$ is the wavevector of the SPW propagating along the planar dielectric/metal boundary, and $\varepsilon_{1}\left(\lambda \right)$ and $\varepsilon_{2}\left(\lambda \right)$ are complex permitivities of the dielectric material and metal, respectively. The same equation can be interpreted as the coupling condition, which has to be fulfilled to excite the SPW. Let us consider that an optical wave incident from the glass prism is coupled into the SPW, as shown in Figure 1a. The SPW is excited under the condition that the tangential component of the wave vector $k_{t}$ of the incident wave is equal to the wave vector $k_{\mathrm{spw}}$ of the SPW. In this case, Equation (1) leads to the phase-matching condition expressed as

(2) n(λ)sinθSPR=ReεAu(λ)na2(λ)εAu(λ)+na(λ),

where n(λ) and $n_{a}\left(\lambda \right)$ are the refractive indices of the prism and analyte (air) at the wavelength λ, respectively, $\varepsilon_{\mathrm{Au}}\left(\lambda \right)$ is the permittivity of gold and $\theta =\theta_{\mathrm{SPR}}$ is the resonance angle of incidence. The sensing is based on resolving a shift of the reflectance dip for a *p*-polarized, transverse magnetic (TM), wave when the refractive index of the analyte is changed. It should be stressed that Equation (1) was obtained for two semi-infinite media [2], but, in the real experimental conditions, the gold layer has a finite thickness. For a finite gold film, the wave vector $k_{\mathrm{spw}}$ in the matching condition (2) is with a correction of $\Delta k_{\mathrm{spw}}$.

**Figure 2:**
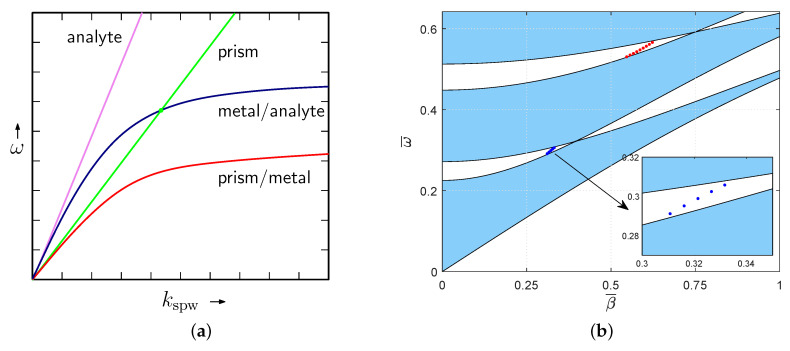
Dispersion of the wavevectors of the light wave in air (violet line) and in the glass prism (green line), the SPP waves at the metal/air interface (blue line), and the prism–metal interface (red line), respectively (**a**); the computed band structure of an infinite periodic MDS for the TM wave (**b**).

To illustrate the SPW excitation under the phase matching condition given by Equation (2), we consider a metallic layer, a glass prism and an analyte (air). The corresponding dispersion of the SPW at the metal–analyte interface is shown in Figure 2a by the blue line which crosses the line for the light wave in the glass prism by the green line so that the SPW is excited. When the light wave in air is considered as shown by the violet line, no crossing is present and the SPR does not occur. Similarly, the SPW is not excited at the prism–metal interface.

### 2.3. Periodic Multilayer Structure

The other way how to excite a surface wave is to substitute the gold layer by a periodic system consisting of dielectric (lossless) bi-layers with high and low refractive indices $n_{a}$ and $n_{b}$. Such a structure forms a photonic crystal of finite thickness, or equivalently the MDS, and, under specific conditions, the BSWs can be excited at the interface between the MDS and external medium [34]. Similarly to the SPW case, the BSW can be excited in the Kretschmann configuration shown in Figure 1b when the appropriate phase matching condition is fulfilled. As in the previous case, the reflectance spectrum plays a crucial role in the sensing. To explain some of its basic features, the band structure of an infinite (perfect) 1DPhC can be used.

The analysis of the electromagnetic field in one bi-layer (considered as a unit cell in the periodic system) using the TMM leads to a so-called translation matrix, whose trace is directly related to the band structure. Using the Bloch approach to the wave propagation in periodic layered media, the resulting eigenproblem yields the translation matrix eigenvalue [73]. For the case of TM waves, this equation has the form:

(3) $$\cos\left(K\Lambda \right)=\cos\left(k_{⊥a}\;a\right)\cos\left(k_{⊥b}\;b\right)-\frac{1}{2}\left(\frac{n_{b}^{2}}{n_{a}^{2}}\frac{k_{⊥a}}{k_{⊥b}}+\frac{n_{a}^{2}}{n_{b}^{2}}\frac{k_{⊥b}}{k_{⊥a}}\right)\sin\left(k_{⊥a}\;a\right)\sin\left(k_{⊥b}\;b\right)\;$$

where Λ=a+b is the bi-layer thickness (spatial period), a,b are the thicknesses of the individual layers, and $k_{⊥a},\;k_{⊥b}$ are the normal components of appropriate wavevectors given as $k_{⊥i}=\sqrt{{\left(n_{i}\omega /c\right)}^{2}-\beta^{2}},\;i=a,\;b$, and β denotes the propagation constant. Equation (3) is the dispersion relation of the infinite periodic system, which couples the propagation constant, frequency, and the Bloch wavenumber *K*. Graphically expressed, the dispersion relation ω=ω(β,K) leads to a band structure: the waves with real *K* falls into the allowed bands (where cos(KΛ)<1) and they can propagate through the periodic structure, whereas the waves with imaginary *K* form forbidden bands (cos(KΛ)>1), usually referred to as photonic band gaps. The band edges correspond to |cos(KΛ)|=1. Even if the waves with imaginary Bloch wavenumber are forbidden in the infinite system, they can exist in the *semi-infinite* or *finite* system. As they are localized near the boundaries of the structure with the surrounding media, they are referred to as Bloch surface waves.

As an example, Figure 2b shows the computed band structure of the infinite 1DPhC consisting of ${\mathrm{TiO}}_{2}/{\mathrm{SiO}}_{2}$ bi-layers with thicknesses a=175nm and b=85nm. The spatial period is Λ=260nm, and TM polarization was assumed. Instead of frequency and propagation constant, the reduced (dimensionless) variables $\bar{\omega }=\frac{\omega }{c}\frac{\Lambda }{2\pi }=\Lambda /\lambda$ and $\bar{\beta }=\beta \frac{\Lambda }{2\pi }$ are used. Usually, the band structure is computed under the assumption that the refractive indices of all media are constant. As the experimental study and the model computation of the reflectance response were performed in a wide spectral interval, the dispersion properties were included into the computation. The model dielectric functions for $n_{a}=n_{{\mathrm{TiO}}_{2}}\left(\lambda \right)$ and $n_{b}=n_{{\mathrm{SiO}}_{2}}\left(\lambda \right)$ were obtained from ellipsometric characterization of the samples [61]. Both models are valid for λ∈〈0.2,1.7〉μm ($\bar{\omega }\in \langle 0.16,\;1.3\rangle$ for given Λ). In Figure 2b, two band gaps can be clearly seen—one of the them is in a near infrared region, and the other one is in a visible region. To show the positions of surface waves in the band structure, the reflectance spectrum was computed using the TMM for a finite MDS comprising 100 bi-layers mentioned in the above text. The TM wave considered to be incident from the glass substrate (the dispersion equation can be found in [61]), and the air was chosen as the analyte. The position of the dips in two spectral regions was traced as the internal incidence angle within the glass was changed. When plotted in the band diagram, Figure 2b, the corresponding points are located in the mentioned band gaps, thus confirming the fact that the appropriate waves are the BSWs.

### 2.4. Reflectance Responses

**Figure 3:**
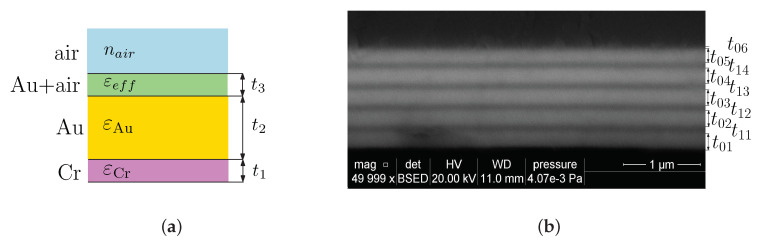
An MPS structure under study (**a**), an SEM photo of an MDS under study (**b**).

To illustrate the SPR phenomenon for a real case, we consider an MPS studied previously [24]. The MPS is shown in Figure 3a and is represented by a system of thin films: an adhesion Cr film, Au film, and the rough Au surface represented by the pseudolayer with the volume fraction *q* = 0.5 of the gold in the thin film. The corresponding thicknesses are $t_{1}$ = 2 nm and $t_{2}$ = 44.8 nm, as specified by producer (Accurion, Goettingen, Germany), and $t_{3}$ = 2 nm, as obtained by an atomic force microscopy analysis [24].

**Figure 4:**
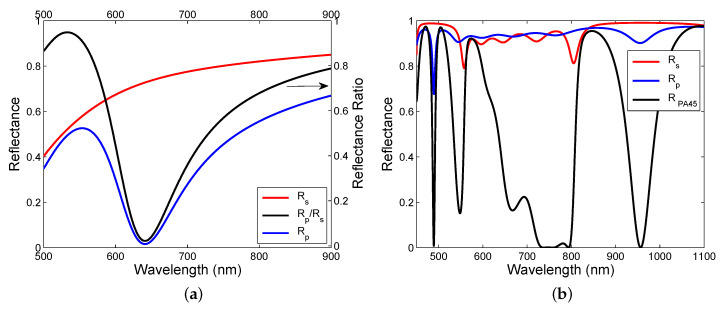
Theoretical spectral reflectances $R_{s}\left(\lambda \right)$ and $R_{p}\left(\lambda \right)$, respectively, for the multilayer structures: the MPS and the reflectance ratio $R_{p}\left(\lambda \right)/R_{s}\left(\lambda \right)$ (**a**), the MDS and the reflectance $R_{\mathrm{PA}45}\left(\lambda \right)$ (**b**). Analyte is air.

In the reflectance evaluations, the refractive index of the external medium (air) is considered to be n=1, taking into account the dispersion of materials of the MPS [24], with dispersion of Au described by dielectric function given by the Drude–Lorentz model with two additional Lorentzian terms

(4) $$\varepsilon_{\mathrm{Au}}\left(\lambda \right)=1-\frac{1}{\lambda_{p}^{2}\left(1/\lambda^{2}+i/\gamma_{p}\lambda \right)}-\sum_{j=1}^{2}\frac{A_{j}}{\lambda_{j}^{2}\left(1/\lambda^{2}-1/\lambda_{j}^{2}\right)+i\lambda_{j}^{2}/\gamma_{j}\lambda },$$

where parameters are specified in Table 1, Figure 4a shows the theoretical reflectances $R_{s}\left(\lambda \right)$ and $R_{p}\left(\lambda \right)$, and the reflectance ratio $R_{p}\left(\lambda \right)/R_{s}\left(\lambda \right)$ as well, as a function of the wavelength λ for the angle of incidence $\theta =37.7^{\circ }$. As can be seen from the figure, a wide dip is obtained for the reflectance of a *p*-polarized wave, or equivalently for the reflectance ratio. The dip is associated with the SPW excitation. The mechanism of SPR dip shift is based on a change in both the refractive index of the external medium (moist air) and the effective refractive index of the pseudolayer.

Similarly, to illustrate the BSWR phenomenon for a real case, we consider an MDS studied previously [61]. The MDS is shown in Figure 3b and is represented by a system of four bilayers (i=j= 1, …, 4) of TiO${}_{2}$/SiO${}_{2}$ with thicknesses $t_{0i}=$ 166.7, 182.8, 179.0, and 183.9 nm, and $t_{1j}=$ 79.9, 89.2, 86.6, and 87.2 nm, respectively, and a termination layer of TiO${}_{2}$ of thickness $t_{05}=$ 177.4 nm with a rough layer of thickness $t_{06}=$ 13.5 nm. A standard approach in the refractive index sensing, which is based on measurement of the reflectance of a *p*- or *s*-polarized wave, a TM or transverse electric (TE) wave, usually fails because resolving the corresponding reflectance dip is not possible due to a shallow resonance dip [63]. Thus, we proposed a new approach [63] utilizing the spectral interference of the polarized waves reflected from the MDS, and the BSW resonance dip can be resolved with maximum depth. The interference is attained when both the polarizer and analyzer are oriented $45^{\circ }$ with respect to the plane of incidence, and the corresponding reflectance $R_{\mathrm{PA}45}\left(\lambda \right)$ is expressed as

(5) $$R_{\mathrm{PA}45}\left(\lambda \right)=\frac{1}{4}\left\{R_{s}\left(\lambda \right)+R_{p}\left(\lambda \right)+2\sqrt{R_{s}\left(\lambda \right)R_{p}\left(\lambda \right)}\cos\left[\delta_{sp}\left(\lambda \right)\right]\right\},$$

where $R_{s}\left(\lambda \right)$ and $R_{p}\left(\lambda \right)$ are reflectances of *s*- and *p*-polarized waves, respectively, and $\delta_{sp}\left(\lambda \right)$ is their phase difference.

To model the spectral responses of the DMS, the TMM was used and the reflectance responses in the Kretschmann configuration with a coupling prism made of BK7 glass were evaluated. In these evaluations, the refractive index of the external medium (air) is considered to be n=1, the dispersion of TiO${}_{2}$ and SiO${}_{2}$ layers of the structure is given by a one-oscillator Sellmeier formula [61]

(6) $$n^{2}\left(\lambda \right)=a+\frac{b\lambda^{2}}{\lambda^{2}-c^{2}}\;$$

where the wavelength λ is in μm and the Sellmeier coefficients for the TiO${}_{2}$ and SiO${}_{2}$ are a=2.7655, b = 2.2 and c=0.26524μm, and a=1.34836, b= 0.75650 and c=0.10683μm, respectively. The extinction coefficients for TiO${}_{2}$ and SiO${}_{2}$ layers are assumed to be $\kappa_{{\mathrm{TiO}}_{2}}=1.6\times 10^{-3}$ and $\kappa_{{\mathrm{SiO}}_{2}}=3.4\times 10^{-4}$, respectively. Figure 4b shows the theoretical reflectance $R_{\mathrm{PA}45}\left(\lambda \right)$ together with reflectances $R_{s}\left(\lambda \right)$ and $R_{p}\left(\lambda \right)$ as a function of the wavelength λ for the angle of incidence $\theta =41.9^{\circ }$. As can be seen from the figure, dips of different widths are obtained within two band gaps for a *p*-polarized wave, a narrow dip within the short-wavelength band gap and a broad and shallow dip within the long-wavelength band gap. The dips are associated with the BSW excitation, and Figure 4b illustrates that the spectral interference of the polarized waves gives dips with maximum depth. The mechanism of BSWR dip shift is based on a change in both the refractive index of the external medium (moist air) and the effective refractive index of the terminated layer.

**Figure 5:**
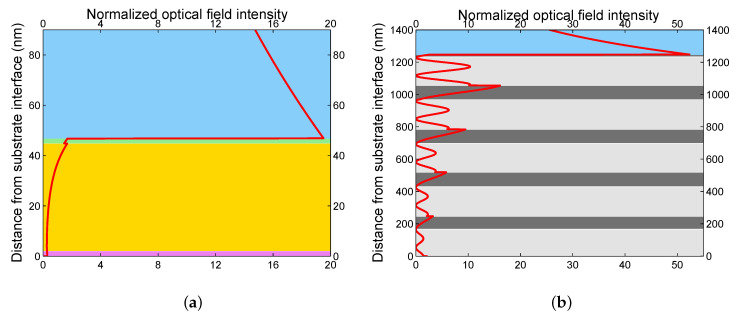
The normalized optical field intensity distribution in the MPS for an angle of incidence of $\theta =37.7^{\circ }$ and λ= 641.6 nm (**a**), and in the MDS for $\theta =41.9^{\circ }$ and λ= 489.2 nm (**b**). TM wave and analyte is air.

**Figure 6:**
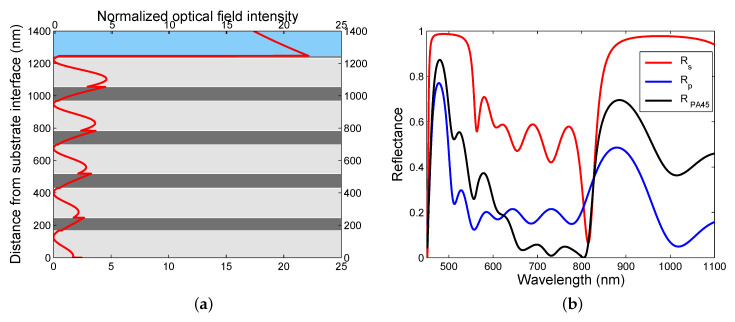
The normalized optical field intensity distribution in the MDS for an angle of incidence of $\theta =41.9^{\circ }$ and λ= 955.4 nm (**a**); Theoretical spectral reflectances $R_{s}\left(\lambda \right)$, $R_{p}\left(\lambda \right)$, and $R_{\mathrm{PA}45}\left(\lambda \right)$ for $\theta =40.5^{\circ }$ (**b**). TM wave and analyte is air.

To confirm the SPR, the optical field intensity ${\left|E\right|}^{2}$ in the MPS divided by ${\left|E_{0}\right|}^{2}$, where $E_{0}$ is the incident *p*-polarized electric field, is shown in Figure 5a for an angle of incidence of $37.7^{\circ }$ and a wavelength of 641.6 nm. Optical field enhancement in the gold layer and the localization of the wave on the surface are apparent from this figure. Similarly, to confirm the BSWR in the short-wavelength band gap (BSW1), the same quantity in the MDS is shown in Figure 5b for an angle of incidence of $41.9^{\circ }$ and a wavelength of 489.2 nm. The SPW exhibits nearly a twenty-fold enhancement, while the BSW1 shows more than a fifty-fold enhancement, and an exponential tail of the wave in the analyte (air) is decaying slower for the SPW than for the BSW1. In addition, to confirm the BSWR in the long-wavelength band gap (BSW2), the normalized optical field intensity in the MPS is shown in Figure 6a for the same angle of incidence and a wavelength of 955.4 nm. The BSW2 exhibits nearly a twenty three-fold enhancement and a slower exponential decay of the wave than for the BSW1. Thus, both the MPS and MDS have the potential to be employed in sensor applications. It should be stressed that four bilayers in the MDS are not optimal as in the case of a semi-infinite MDS, as demonstrated by different shapes of the field envelopes within the MDS shown in Figure 5b and Figure 6a. Fortunately, the sensing applications are still possible.

Figure 6b shows the theoretical spectral reflectances $R_{s}\left(\lambda \right)$ and $R_{p}\left(\lambda \right)$, together with the reflectance $R_{\mathrm{PA}45}\left(\lambda \right)$ for the angle of incidence $\theta =40.5^{\circ }$. The figure clearly demonstrates the disappearance of the BSW resonances and the reflectance decreases for both $R_{p}\left(\lambda \right)$ and $R_{\mathrm{PA}45}\left(\lambda \right)$ when the angle of incidence becomes less than the critical angle.

## 3. Experimental Setup

**Figure 7:**
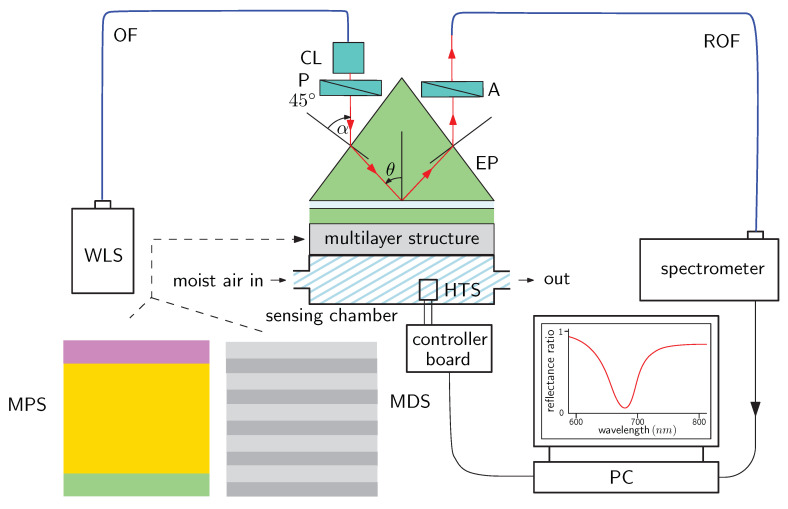
Experimental setup: a multilayer structure in the Kretschmann configuration; white-light source (WLS), optical fiber (OF), collimating lens (CL), polarizer (P), equilateral prism (EP), analyzer (A), read optical fiber (ROF), humidity and temperature sensor (HTS), dielectric plasmonic structure (MPS), multilayer dielectric structure (MDS), and personal computer (PC).

An experimental setup used to measure the reflectance response of multilayer structures with the RH change, or equivalently with the change in the refractive index of moist air, is shown in Figure 7. It comprises a white-light source (WLS) (halogen lamp HL-2000, Ocean Optics, Dunedin, FL, USA) terminated by launching optics, to which an optical fiber (OF) with a collimating lens (CL) are connected. The beam from the collimating lens is 1 mm diameter and next is a linear polarizer (P) (LPVIS050, Thorlabs, Newton, MA, USA). Its orientation is $45^{\circ }$ with respect to the plane of incidence, and both polarization components, *p* and *s*, are generated. The beam is coupled to a multilayer structure on a glass slide using an equilateral prism (EP). The light reflected from the multilayer structure passes through a linear analyzer (A) (LPVIS050, Thorlabs) oriented $0^{\circ }$ and $90^{\circ }$, or $45^{\circ }$ with respect to the plane of incidence so that the reflectances $R_{p}\left(\lambda \right)$ and $R_{s}\left(\lambda \right)$, or $R_{\mathrm{PA}45}\left(\lambda \right)$ are measured. A read optical fiber (ROF) (M15L02, Thorlabs) is used to launch the light directly into a spectrometer (USB4000, Ocean Optics), which is connected via USB to a personal computer (PC).

In the case of the MPS, which was prepared on the SF10 glass slide, the slide was attached to the EP made of the SF10 glass (Accurion, Goettingen, Germany) by a thin film of index-matching fluid (Cargille, Cedar Grove, NJ, USA, $n_{D}$ = 1.730). In the case of the MDS, which primarily served as interference filter, and it was prepared by a technique of sputtering (Meopta, Přerov, Czech Republic), the glass slide with the MDS [61] was attached to the EP made of the BK7 glass (Ealing, South Natick, MA, USA) with index-matching fluid (Cargille, $n_{D}$ = 1.516).

The MPS or MDS is attached via O ring to a sensing chamber (volume approximately 22 mL) hosting an electrical humidity and temperature sensor (HTS) (HTU21D, Arduino, Ivrea, Italy) connected to a controller board (Arduino UNO). A part of the setup for the adjustment of the relative humidity of air consists of a humidifier [74] and a two-line peristaltic pump (BT100M, 2xYZ1515x, Baoding Chuang Rui Precision Pump Co., Ltd.). The first line of the peristaltic pump is connected to the input of the humidifier, and the second line with the output of the humidifier is connected to the chamber. The humidifier is represented by a bottle of a 400 mL volume with 300 mL of distilled water. A constant air flux is sent through the water in the bottle and controlling the flow of moist air from the bottle in one line by means of the peristaltic pump regulator, the relative humidity of air in the chamber can be varied approximately in a range of 18–82 %RH. The lowest relative humidity of air is attained when outer dry air (∼18 %RH) flows directly through the chamber. The highest RH can be controlled by the flow of moist air and thus depends on the peristaltic pump used.

## 4. Experimental Results and Discussion

**Figure 8:**
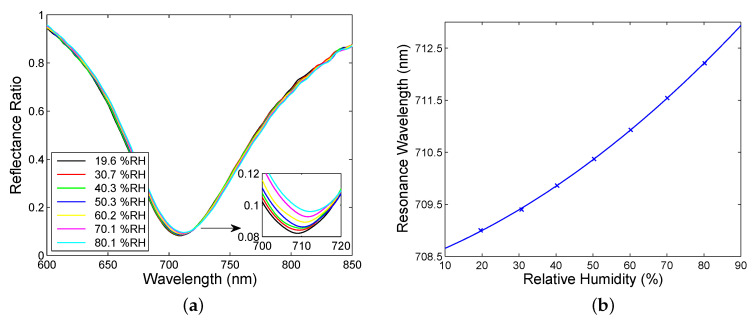
Spectral reflectance ratio $R_{p}\left(\lambda \right)/R_{s}\left(\lambda \right)$ for different values of the RH of moist air measured for the MPS (**a**). Resonance wavelength as a function of the RH of moist air with a polynomial fit (**b**).

To demonstrate that both multilayer structures have potential to be employed in sensor applications, the setup shown in Figure 7 and the wavelength interrogation were utilized in the relative humidity measurements at a temperature of 22.5 ${}^{{}^{\circ}}$C. The responses of the multilayer structures were compared from the point of view of the sensitivity and FOM, respectively. First, the SPR based humidity measurements were performed, and, in Figure 8a, the measured reflectance ratio $R_{p}\left(\lambda \right)/R_{s}\left(\lambda \right)$ as a function of the wavelength λ for the external angle of incidence $\alpha =41.6^{\circ }$ (see Figure 7) and the relative humidity of air ranging from 19.6 %RH to 80.1 %RH are also shown. This figure illustrates the excitation of the SPW manifested by a relatively wide and sufficiently pronounced dip near a wavelength of 710 nm. The wide dip is due to the losses of the gold film in the MPS and the full width at half maximum (FWHM) of the dip is about 142 nm, and it is nearly constant with the changes in the relative humidity of air. The position of the dip, the resonance wavelength, is shifted, and the shift is enlarged as the relative humidity of air increases, as shown in Figure 8b.

**Figure 9:**
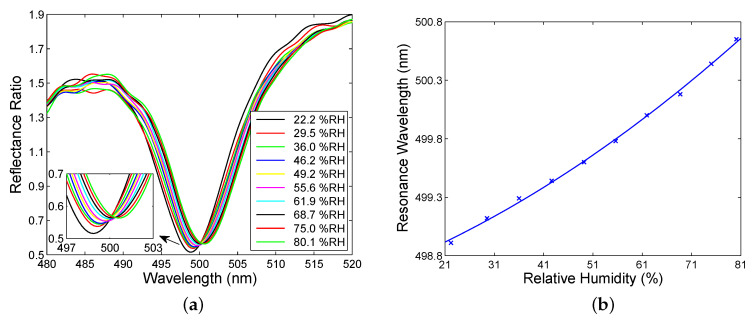
Spectral reflectance ratio $R_{\mathrm{PA}45}\left(\lambda \right)/R_{s}\left(\lambda \right)$ for different values of the RH of moist air measured within the short-wavelength band gap (BSW1) (**a**). Resonance wavelength as a function of the RH of moist air with a polynomial fit (**b**).

Next, the BSWR based humidity measurements were performed, utilizing an approach presented above, which is based on the spectral interference of *s*- and *p*-polarized waves reflected from the MDS. Thus, Figure 9a shows the wavelength dependence of the measured reflectance ratio $R_{\mathrm{PA}45}\left(\lambda \right)/R_{s}\left(\lambda \right)$ for the external angle of incidence $\alpha =28.9^{\circ }$ and the relative humidity of air ranging from 22.2 %RH to 80.1 %RH. This figure illustrates the excitation of the BSW manifested by a sufficiently pronounced dip near a wavelength of 500 nm. It is interesting to note that the large jitter at the beginning and end of the spectrum is caused by the lower signal in these regions than that near resonance with the optical field enhancement. The dip is present within the short-wavelength band gap (BSW1), and the depth of the dip decreases with the relative humidity of air. The resonance wavelength shift is enlarged as the relative humidity of air increases, as shown in Figure 9b, and the FWHM of the dip is approximately 9.3 nm.

**Figure 10:**
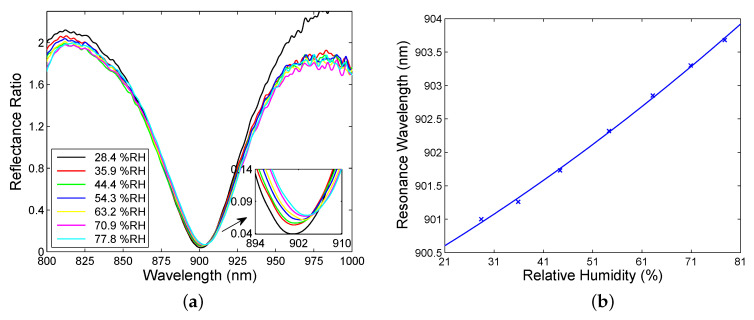
Spectral reflectance ratio $R_{\mathrm{PA}45}\left(\lambda \right)/R_{s}\left(\lambda \right)$ for different values of the RH of moist air measured within the long-wavelength band gap (BSW2) (**a**). Resonance wavelength as a function of the RH of moist air with a polynomial fit (**b**).

The BSW excitation is also manifested within the long-wavelength band gap (BSW2) when the angle of incidence $\alpha =23.6^{\circ }$. This is demonstrated in Figure 10a depicting a dip near a wavelength of 900 nm with a nearly maximum depth. Contrary to the BSW1, the dip is substantially broader with an FWHM of approximately 60 nm. The large width of the dip is done mostly by a small number of bilayers of TiO${}_{2}$/SiO${}_{2}$ in the structure [47]. The depth of the dip decreases with the relative humidity of air and the position of the dip shifts toward longer wavelengths as the relative humidity of air increases, as shown in Figure 10b. Moreover, when the relative humidity of air decreased, no hysteresis was revealed in a quick response to the relative humidity changes. This is due to sensing principle based on surface waves and the dielectric termination layer characterized by both mechanical and chemical stability.

It is also desirable to evaluate the sensitivity $S_{\mathrm{RH}}$ to the relative humidity, defined as the change of the position of the dip $\delta \lambda_{r}$ with respect to the change in the relative humidity δRH of moist air

(7) $$S_{\mathrm{RH}}=\frac{\delta \lambda_{r}}{\delta \mathrm{RH}}.$$

**Figure 11:**
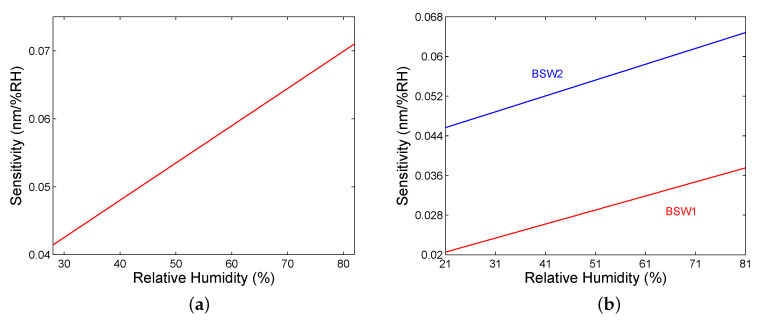
Sensitivity as a function of the RH of moist air for SPR (**a**), and for two BSWs (**b**).

For the SPR based sensor, the resonance wavelength shift versus the RH can be well fitted by a second-order polynomial as shown in Figure 8a, and the sensitivity $S_{\mathrm{RH}}$ exhibits a linear dependence on the RH, as depicted in Figure 11a. The sensitivity is in a range of 0.042–0.072 nm/%RH. Similarly, the resonance wavelength shift functions for the BSWRs can be well fitted by second-order polynomials as shown in Figure 9b and Figure 10b, and the sensitivity $S_{\mathrm{RH}}$ exhibits in both cases a linear dependence on the RH, as depicted in Figure 11b. Within the short-wavelength band gap of the BSWR based sensor, the sensitivity is in a range of 0.021–0.038 nm/%RH, and, within the long-wavelength band gap, it is enhanced and changes in a range of 0.046–0.065 nm/%RH. Comparing these values with those of optical RH sensors as listed in Table 2, highly sensitive RH sensors utilizing the BSWR are possible. In addition, fiber-optic RH sensors of substantially higher sensitivities are available [75,76].

It would also be desirable to express the sensitivity to the refractive index (RI), but changes in the RI are different from those for a standard measurement [82,83] giving for a 100 %RH change the RI change of 9.4 × 10${}^{-7}$. The changes in the RI of the external medium are not known and they are affected by the adsorption of water molecules on the rough surface of the MDS [84], and this is similar to porous silicon having a large internal surface area for adherence of analytes [38,59]. A shift in the resonance is affected by a change in the refractive indices of the external medium (moist air) of the sensor and its termination material. Actually, water vapor adsorption and capillary condensation take place in pores of nanometer size, and, as the pores are filled with water, the effective refractive index of the terminated layer is being increased [84]. The theoretical analysis of the response of the MDS can be performed when the roughness (porosity) of the termination layer is known.

The limit of detection for the RH (${\mathrm{LOD}}_{\mathrm{RH}}$), defined as ${\mathrm{LOD}}_{\mathrm{RH}}=\Delta \lambda /S_{\mathrm{RH}}$, can also be expressed. If the precision Δλ of resolving the resonance wavelength is 0.01 nm, the ${\mathrm{LOD}}_{\mathrm{RH}}$ reaches 0.15 %RH for the BSW2. The LOD can also be defined as the minimal amount of change in the RH that can be detected by the sensor, and it is expressed through its standard deviation [7].

We can also evaluate the FOM of the RH measurement given by a similar relation to the FOM of the refractive index measurement [43,47]

(8) $$\mathrm{FOM}=S_{\mathrm{RH}}\frac{D}{W},$$

where *D* and *W* are the depth and FWHM of the dip, respectively. In the case of the SPR measurement, D= 0.918 and the FOM is up to 4.7$\times 10^{-4}$ %RH${}^{-1}$. The BSWR based measurements are characterized by substantially narrower resonance dips and thus, for the short-wavelength band gap with D= 0.737, the FOM is as high as 3.0$\times 10^{-3}$ %RH${}^{-1}$. Similarly, for the long-wavelength band gap with D= 0.983, the FOM reaches 1.1$\times 10^{-3}$ %RH${}^{-1}$.

## 5. Conclusions

In this paper, a highly sensitive measurement of the relative humidity of moist air, based on both the SPW and BSW excitation, and the wavelength interrogation have been presented. The relative humidity measurement was performed for an MPS of SF10/Cr/Au and for an MDS comprising four bilayers of TiO${}_{2}$/SiO${}_{2}$ with a rough termination layer of TiO${}_{2}$. We revealed for moist air that the SPW excitation shows up as a wide dip in the reflectance of a *p*-polarized wave, and the SPR effect is characterized by sensitivities up to 0.072 nm/%RH. Similarly, employing an effective approach based on the spectral interference of polarization waves, we revealed that the BSW resonances, which are of a sufficient depth and magnitude, are comparable with the SPR resonances. The BSW resonances were resolved within two band gaps of the MDS, and the measured sensitivities were up to 0.065 nm/%RH. We also revealed that the SPR based relative humidity measurement is with the FOM of 4.6 × 10${}^{-4}$ %RH${}^{-1}$, while, for BSWR based measurements, FOM reached 3.0 × 10${}^{-3}$ %RH${}^{-1}$.

The obtained results demonstrate that the BSWR based sensor employing the available MDS has a similar sensitivity as the SPR based sensor, but outperforms it in the FOM. BSW based sensors employing dielectrics thus represent an effective alternative with a number of advantages, including better mechanical and chemical stability than metal films used in SPR sensing. Thus, wider adoption of BSW based sensors is possible as has been demonstrated by an ultra-low-cost 3D-printed optical sensor [85]. In addition, we revealed that, due to mechanical and chemical stability of the MDS employed in the sensor, no hysteresis in the relative humidity measurement was revealed. Finally, owing to a quick response of the MDS to the relative humidity changes, the sensor has the potential to be applied in real-time measurements, including monitoring human breath.

## Figures and Tables

**Table 1:** Parameters of dielectric function of Au [24].

Drude Term	Value	Oscillator 1	Value	Oscillator 2	Value
Parameter		Parameter		Parameter	
$\varepsilon_{\infty }$	1	$A_{1}$	8.88	$A_{2}$	1.70
$\lambda_{p}$ (nm)	130.77	$\lambda_{1}$ (nm)	255.5	$\lambda_{2}$ (nm)	660.67
$\gamma_{p}$ (nm)	6608.3	$\gamma_{1}$ (nm)	−29.73	$\gamma_{2}$ (nm)	−819.68

**Table 2:** Comparison of different optical RH sensors.

Material	Method	RH Range	Sensitivity (nm/%RH)	Ref.
polymer coating	whispering gallery mode resonance	0–60%	0.013	[77]
agarose gel	guided mode resonance	20–80%	0.150	[78]
porous thin film	photonic crystal mode resonance	11–84%	0.296	[79]
indium tin oxide	lossy mode resonance	65–90%	0.212	[80]
copper oxide	lossy mode resonance	30–90%	0.636	[81]
